# Structural elucidation, biological significance and computational approach of Copper(ii), Nickel(ii) and Cobalt(ii) with bidentate schiff base of N-(Napthalene-1-ylmethylene)isonicotinohydrazide

**DOI:** 10.1038/s41598-025-23208-3

**Published:** 2026-01-29

**Authors:** Md. Ashraful Islam, Faria Tasnim, Md. Toukir Biswas, Md. Sajib Hossain, Md. Eram Hosen, Md. Robiul Islam, Md. Ali Asraf, Md. Faruk Hossen, Al-Anood M. Al-Dies, Md. Kudrat-E-Zahan, Zsolt Tóth, Magdi E. A. Zaki

**Affiliations:** 1 Department of Chemistry, University of Rajshahi, Rajshahi, 6205 Bangladesh; 2 Department of Genetic Engineering and Biotechnology, University of Rajshahi, Rajshahi, 6205 Bangladesh; 3 Department of Applied Chemistry and Chemical Engineering, University of Rajshahi, Rajshahi, Bangladesh; 4 Biomedical Science and Engineering, College of Medicine and Dentistry, James Cook University, Townsville, Australia; 5 Department of Chemistry, Umm Al-Qura University, Al-Qunfudah University College, Mecca, Saudi Arabia; 6 Faculty of Wood Engineering and Creative Industries, University of Sopron, Sopron, Hungary; 7 Department of Chemistry, College of Science, Imam Mohammad Ibn Saud Islamic University, Riyadh, 15, Saudi Arabia

**Keywords:** Schiff base, Metal complexes, Antibacterial activity, Antioxidant activity, Molecular docking, DFT analysis, Biochemistry, Computational biology and bioinformatics, Drug discovery, Structural biology

## Abstract

The increasing prevalence of antibiotic-resistant bacteria and oxidative stress-related diseases underscores the need for novel therapeutic agents with potential dual functionality. In this research, a schiff base ligand, N-(Napthalene-1-ylmethylene)isonicotinohydrazide, was synthesized and complexed with Copper(II), Nickel(II), and Cobalt(II) ions. Characterization of the compounds using various spectroscopic and analytical techniques confirmed successful complex formation and structural stability. Antibacterial testing through the disc diffusion method revealed that the Ni(II) complex exhibited the highest antibacterial activity, with significant inhibition against *Staphylococcus aureus*, *Bacillus subtilis*, *Escherichia coli*, and *Shigella flexneri*. The antioxidant activity, evaluated via DPPH radical scavenging, showed that the Cu(II) complex was the most effective with an IC_50_ value of 187.81 ± 1.42 µg/mL. Molecular docking studies against DNA gyrase (PDB ID: 7P2M) predicted the Ni(II) complex as the best binder (–9.9 kcal/mol), suggesting strong initial affinity. Complementary molecular dynamic simulations further demonstrated that Cu(II) and Co(II) complexes maintained the most stable protein-ligand interactions under dynamic conditions, highlighting their potential as robust inhibitors. ADMET predictions indicated favorable pharmacokinetic and toxicity profiles, though recognized as preliminary. DFT calculations confirmed stable geometries and showed a reduction of the HOMO–LUMO energy gap from 4.21 eV (ligand) to 2.85 eV (Ni complex), consistent with enhanced reactivity and biological activity. Mapping of electron density and atomic charge analysis identified potential nucleophilic attack sites, reinforcing the complexes’ therapeutic potential in combating bacterial infections and oxidative stress.

## Introduction

Bacterial infections pose significant threats to human health, often compromising the immune system and exacerbating various medical conditions. The increasing prevalence of antibiotic resistance is a concerning global health issue. In 2023, over 1.05 million deaths were associated with antimicrobial resistance (AMR) and 250,000 individuals died directly due to AMR, with projections indicating millions more could be affected^1^. Many existing antibiotics are ineffective against bacterial infections due to factors such as biofilm formation, strain variation, and interactions with other pathogens. Additionally, oxidative stress can worsen the health of patients already battling bacterial infections, leading to more severe conditions^2^. Oxidative stress occurs when oxygen radical production exceeds antioxidant capacity, damaging essential cell molecules and leading to various diseases. In serious cases like cancer, ocular degeneration, and neurodegenerative diseases, oxidative stress is the original factor. In infectious diseases, oxidative stress occurs secondary to the initial disease and plays a significant role in immune or vascular complications. Dealing with these challenges requires the development of novel drugs capable of combating both infections and oxidative stress.

Schiff bases are a major class of ligands in co-ordination chemistry as well as integral to both medicinal and material chemistry for an extended period of time. These organic ligands are derived through the 1:2 stepwise condensations of carbonyl compounds with alkyl or aryl diamines, alongside various aldehyde or ketone under controlled conditions. They can stabilize metal ions across various oxidation states, facilitated by the nitrogen lone pair of electrons within their azomethine (-N = CH-) bonding structure. The schiff base complexes of transition metals are currently gaining considerable attention due to their wide array of biological and pharmaceutical properties, including antibacterial, antioxidant, anti-fungal, anti-proliferative, anti-malarial and anticancer activities and so on^3–5^. However, many existing Schiff base metal complexes still face limitations such as poor stability under physiological conditions, potential cytotoxicity, or limited selectivity, necessitating the design of safer and more effective derivatives. The medicinal efficacy of transition metal complexes depends on the specific metal ions and ligands used, with metal ions enhancing both the therapeutic effects of the drug and the effectiveness of the organic ligands.

Transition metal complexes with schiff base ligands can exhibit enhanced antibacterial and antioxidant properties, efficiently removing disease-linked free radicals and outperforming isolated schiff base ligands^6–8^. The enhanced antioxidant and antimicrobial properties of the complex are caused by the schiff base ligand’s chelating behavior upon complexation with metals. Consequently, schiff base metal complexes exhibit potential dual-function activity, serving as both antimicrobials and antioxidants^4,6^. Copper (Cu), nickel (Ni), and cobalt (Co) in their divalent forms are well known for their inherent antibacterial and antioxidant properties. Additionally, these metal ions have been well-documented for their favorable coordination geometries, strong binding affinity to ligands, and superior biological activities compared to other transition metals^9–11^. In this study, we report physicochemical characterization of N-(Napthalene-1-ylmethylene)isonicotinohydrazide ligand and its schiff base complexes of Cu(II), Ni(II) and Co(II) along with antibacterial activities against two Gram-positive and two Gram-negative bacteria as well as their antioxidant activity. DNA Gyrase is a type II topoisomerase that is essential for DNA replication, transcription, and chromosome segregation in both Gram-positive and Gram-negative bacteria. Notably, the absence of DNA gyrase in human cells reduces the risk of host cell toxicity making it a prime antibacterial target^12^. Several clinically used antibiotics, such as fluoroquinolones, act by inhibiting DNA Gyrase, making it a strategic target for the development of novel antibacterial agents^13^. Therefore, molecular docking investigation was conducted with DNA Gyrase (PDB ID: 7P2M) for the assessment of interactions of N-(Napthalene-1-ylmethylene)isonicotinohydrazide ligand and its metal complexes. While numerous schiff-based ligands and their metal complexes have been explored for targeting DNA Gyrase in drug discovery using various bioinformatics tools and software, N-(naphthalene-1-ylmethylene)isonicotinohydrazide and its bidentate complexes with Cu, Ni, and Co for targeting DNA Gyrase is novel and has not been computationally or biologically explored. Based on these considerations, the present study aimed to synthesize and characterize the schiff base ligand N-(Napthalene-1-ylmethylene)isonicotinohydrazide and its Cu(II), Ni(II), and Co(II) complexes, and to investigate their antibacterial and antioxidant activities. In addition, computational approaches including molecular docking, ADMET predictions, and DFT calculations were employed to correlate their biological activities with structural and electronic properties. *E. coli* K12 strain was selected as the representative bacterium as it is a widely used model organism with a fully sequenced and extensively characterized genome to assess the inhibitory impact of the synthesized compounds through a computational approach. Its DNA Gyrase enzyme has been structurally resolved at high resolution (PDB ID: 7P2M), making it an ideal and accessible target for computational interaction studies. Additionally, DNA Gyrase in *E. coli* K12 shares high structural and functional homology with the enzyme in other clinically relevant bacterial species, allowing predictive insights into antibacterial potential across multiple pathogens^14,15^. Thus, this comprehensive strategy introduces a novel class of schiff base metal complexes with promising functionality for drug development.

## Experimental

### Resources and reagents

Commercially obtained chemicals were used directly out of the container, without further purification. All of the chemicals used in this study—including 1-naphthaldehyde, isoniazid, copper(II) acetate tetrahydrate, cobalt(II) acetate tetrahydrate, nickel(II) acetate tetrahydrate, 1,1-diphenyl-2-picrylhydrazyl (DPPH), and DMSO (dimethyl sulfoxide)—were purchased from Sigma-Aldrich (India) with stated purities of ≥ 98%. The analytical-grade organic solvents utilized in this study, such as ethanol and DMF (dimethylformamide), were obtained from Merck with purities of ≥ 99.5% and were used without further purification.

### Instrumentation

We used multiple analytical techniques to characterize the ligands and their metal complexes. Melting points were determined using an AZ6512 electrothermal melting point apparatus. Infrared (IR) spectra were recorded on a FTIR-8400 SHIMADZU infrared spectrophotometer using potassium bromide (KBr) discs. UV spectra were measured at a concentration of 5 × 10⁻⁴ M using Shimadzu Double Beam spectrophotometers (UV 1200 and UV-1650PC). The magnetic moments of the solid complexes were assessed with a SHERWOOD SCIENTIFIC Magnetic Susceptibility Balance. Electrical conductance of 10⁻³ M solutions in DMSO was measured using a platinum electrode connected to a WPACM35 conductivity meter. Elemental analysis was carried out with a CHNS/O Analyzer (2400 Series II - PerkinElmer) content in deacetylated chitosan (DCS) to determine the carbon, hydrogen, and nitrogen in the synthesized ligand and their metal complexes.

### Ligand N-(Napthalene-1-ylmethylene) synthesis

The ligand (L) was synthesized via a condensation reaction between 1-naphthaldehyde (20 mmol, 2.1 mL) and isoniazid (10 mmol, 1.08 g). Isoniazid was dissolved in heated ethanol, while 1-naphthaldehyde was dissolved in 20 mL of ethanol. The two solutions were mixed, and the reaction mixture was refluxed for 6–7 h. Upon cooling, a white crystalline product formed, which was collected by filtration and washed sequentially with ethanol, acetone, and diethyl ether. The final product was dried over anhydrous CaCl_2_ in a vacuum desiccator. The ligand was further purified by recrystallization from ethanol, affording a crystalline product of high purity. Characterization revealed that the ligand was soluble in DMSO, ethanol, methanol, and chloroform.

### Metal complex synthesis

Warm ethanolic solution (20 mL) was combined with N-(Napthalene-1-ylmethylene) (0.55 g, 2 mmol) and corresponding metal acetate salts solution of Cu (0.199 g, 1 mmol), Ni (0.248 g, 1 mmol), and Co (0.262 g, 1 mmol) and refluxed for 5–6 h while stirring continuously. The reaction produced colorful precipitates. After cooling, the precipitates were collected by filtration, dried over anhydrous CaCl_2_, and washed thoroughly with ethanol and distilled water to remove any unreacted starting materials. The purity of the complexes was confirmed by thin-layer chromatography (TLC), and their solubility was tested in DMF and DMSO. Finally, the corresponding metal complexes were obtained as solid products and dried in a vacuum desiccator prior to further characterization.

**[N-(naphthalen-1-ylmethyleneisonicotinhydeazide]** C_**17**_**H**_**13**_**N**_**3**_**O (L)**:

White crystals (yield 80%), M.P. 115–117 °C; IR (KBr, ν cm − 1): 3298, 3350 (NH) and 1692 (C = O amide); 1556 (C = N, azomethine), Elemental analysis **(**C_17_H_13_N_3_O) (275.311 g/mol): C, 74.17; H, 4.76; N, 15.26; O, 5.81.

**bis((2-((E)-naphthalen-1-ylmethylene)hydrazineyl)(pyridin-4-yl)methoxy)copper(II) (CuL)**:

Green (yield 75%), M.P.: ˃300 °C; IR (KBr, ν cm − 1): 3434(OH),1354 (C-O); 704((Cu-O), and 657 (Cu-N). Elemental analysis C_34_H_24_CuN_6_O_2_ (612.152 g/mol): C, 66.71; H, 3.95; N, 13.73; Cu, 10.38; O, 5.23.

**bis((2-((E)-naphthalen-1-ylmethylene)hydrazineyl)(pyridin-4-yl)methoxy)nickel(II) (NiL)**:

Green (yield 75%), M.P.: ˃300 °C; IR (KBr, ν cm − 1): 3412(OH), 1372 (C-O); 703(Ni-O), 656 (Ni-N). Elemental analysis C_34_H_24_NiN_6_O_2_ (607.299 g/mol): C, 67.24; H, 3.98; N, 13.84; Ni, 9.66; O, 5.27.

**bis((2-((E)-naphthalen-1-ylmethylene)hydrazineyl)(pyridin-4yl)methoxy)cobalt(II) (CoL)**:

Brown (yield 75%), M.P.: ˃300 °C; IR (KBr, ν cm − 1): 3430 (OH), 1383 (C-O); 702 (Co-O), 657 (Co-N). Elemental analysis C_34_H_24_CoN_6_O_2_ (607.539 g/mol): C, 67.22; H, 3.98; N, 13.83; Ni, 9.70; O, 5.27.

### Antibacterial study

The antibacterial activity of the ligand and its synthesized complexes against four bacterial strains was evaluated using the Kirby-Bauer disc diffusion method with slight modifications^16^. The susceptibility of gram-positive bacteria (*Bacillus cereus* and *Staphylococcus aureus*) and gram-negative bacteria (*Escherichia coli* and *Shigella flexneri*) to these compounds was tested using Mueller-Hinton agar (HiMedia, India). Sterile Whatman No. 1 filter paper discs (6 mm) were impregnated with the test compounds after being dissolved in DMSO at the concentration of 50 µg/ml. The discs were placed on bacteria inoculated petridishes and incubated overnight at 37 °C. Kanamycin was used as a standard. The diameter of inhibition zone surrounding each disc was measured in mm (millimeter). All measurements were replicated three times.

### Antioxidant study

We evaluated the antioxidant activity of the schiff base ligand and its metal complexes using a quantitative DPPH assay. The experimental procedures were performed with slight modifications based on a previously reported method^7^. Samples (1 mg/ml) were prepared as stock solutions in DMSO, from which concentrations of 20, 40, 60, 80, and 100 µg/ml were obtained by serial dilutions. The DPPH solution was combined with the solutions in an aliquot at a ratio of 3:2. For thirty minutes, these mixtures were left at room temperature in the dark. The absorbance at 516 nm was used to calculate the DPPH inhibition percentages (%). Plotting the percentage of inhibition against the concentration yielded the IC_50_ value. BHT was used in the same concentration range as a positive standard. The percentage of scavenging activity at different concentrations of the respective sample fractions was calculated using the following formula:

$${\% \text{ Radical scavenging activity }} = {\text{ }}\left( {{{\mathrm{A}}_{\mathrm{c}}} - {{\mathrm{A}}_{\mathrm{s}}}} \right)/{{\mathrm{A}}_{\mathrm{c}}} \times {\text{ }}100$$

Where,

A_c_ = Absorbance of the control

A_s_= Absorbance of the sample

### Statistical analysis

All experiments were carried out in triplicate and the results are given as mean ± standard deviation (SD). Correlation analysis was carried out for the antioxidant activity method using Excel-2016.

## Theoretical calculation

### Ligand Preparation

The synthesized ligand and its metal complexes revealed drug-like characteristics following ADMET (Absorption–Distribution–Metabolism–Excretion-Toxicity) analysis. These complexes were then subjected to further in silico analysis. ChemDraw software was used to draw the 3D structures and saved in SDF (Structured Data File) format.

### Protein preparation

The x-ray crystallographic structure of DNA gyrase (PDB ID: 7P2M) was acquired from the RCSB PDB website. Using Discovery Studio (version 21.1.0.0), the structure was refined, cleaned by all removing heteroatoms and water molecules. The potential energy of protein was minimized and optimized using the SwissPDB Viewer program’s GROMOS96 43b1 force field.

### Molecular docking

We performed molecular docking using PyRx software (version 0.8) using a previously employed methodology with a few minor modifications^17^. The protein structure was converted into a macromolecule. Subsequently, the schiff base ligand and its metal complexes were then imported and energy minimized using UFF (universal force field) and optimization algorithms. Following conversion into PDBQT format, we performed the docking computations. Discovery Studio was used to analyze the binding interactions and poses.

### ADMET analysis

The ADMET characteristics of the schiff base ligand and its metal complexes were evaluated using SwissADME (http://www.swissadme.ch/), pkCSM (https://biosig.lab.uq.edu.au/pkcsm/) and Protox-3 (https://tox.charite.de/protox3/) server. The investigation of ADMET analysis helps identify and refine potential therapeutic candidates. Comprehending these attributes is essential in the initial phases of computer-aided drug design (CADD) to guarantee the efficacy and soundness of possible therapeutic candidates.

### Molecular dynamics

Molecular dynamics (MD) simulations were performed using the AMBER14 force field implemented in YASARA Dynamics software (version 19.12.4)^18^. Initially, the hydrogen bond network was optimized, and the docked complexes were refined. Energy minimization was then carried out with the steepest descent method in a TIP3P water solvation model^19^. The system was set at a density of 0.997 g/L, temperature of 25 °C, and pressure of 1 atm. To mimic physiological conditions, simulations were maintained at 310 K, pH 7.4, with 0.9% NaCl for system neutralization. A time step of 1.25 fs was applied, while long-range electrostatic interactions were computed using the Particle Mesh Ewald (PME) method with an 8.0 Å cutoff radius^20^. Each simulation was run for 100 ns, with trajectory snapshots recorded every 100 ps. Post-simulation analyses included evaluation of root-mean-square deviation (RMSD), solvent-accessible surface area (SASA), radius of gyration (Rg), root-mean-square fluctuation (RMSF), and hydrogen bond dynamics.

### DFT analysis

GaussView 5.0 was utilized to study the optimized geometry of all the synthesized compounds. The DFT (Density Functional Theory) evaluations were performed using the B3LYP level that combines Hartree-Fock exchange with density functional approximations for improved accuracy. For the ligand, 6-311G(d, p) basis set was employed that offers a triple zeta quality with polarization functions^21^. Conversely, 6-31G(d, p) basis set was utilized for the metal complexes for balancing computational efficiency and accuracy for metal containing system^22^. The computational analysis was conducted using GAUSSIAN 09 and Schrödinger 2022-4 software.

## Results and discussion

### Molar conductivity, electronic spectra, and magnetic moment

**Fig. 1:**
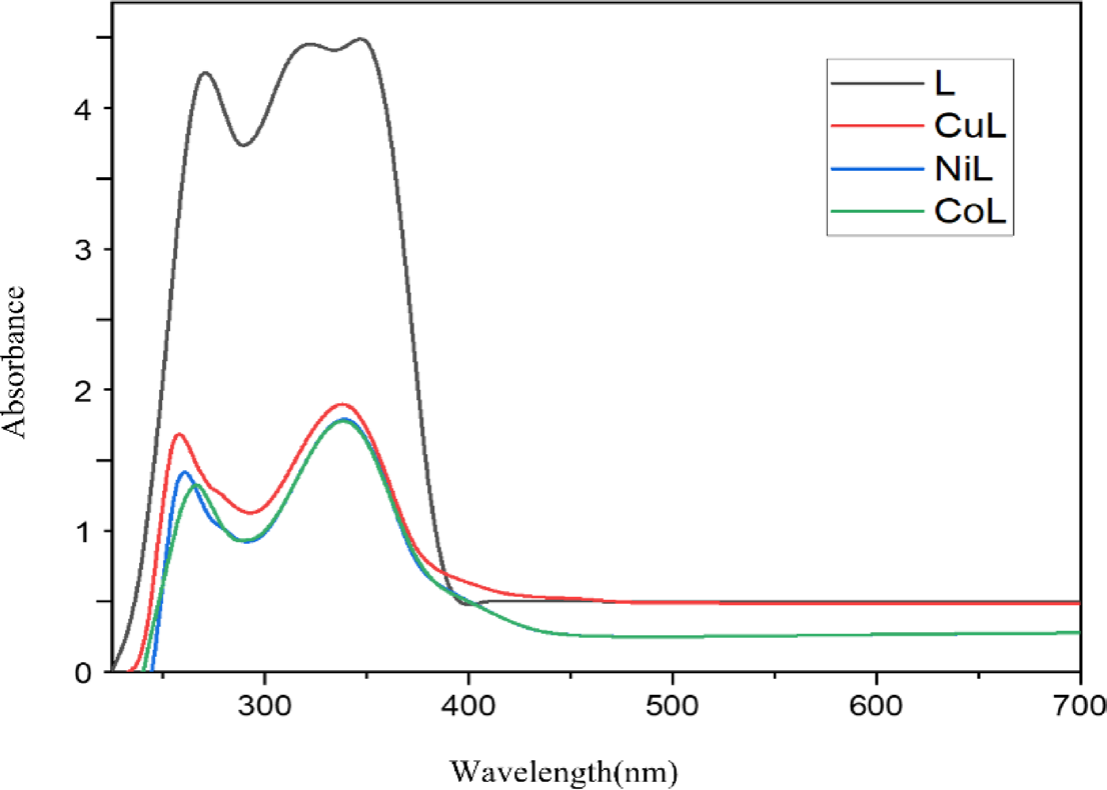
Electronic spectra of synthesized ligand and its metal complexes.

Figure 1; Table 1 present the UV–Vis absorption spectra of the free ligand and its metal complexes (CuL, NiL, and CoL), recorded in DMSO at room temperature over the 200–800 nm range. The spectra exhibited distinct electronic transitions which confirmed successful coordination of the ligand to the metal centers and provided insights into the electronic environment and probable geometry around each metal ion. The spectra showed distinct electronic transitions, which confirms ligand coordination with the metal ions. These spectral shifts also specified changes in the electronic environment and proposed the possible geometry around each metal center. The ligand showed absorption bands at 270 nm and 348 nm attributed to π→π* and n→π* transitions, respectively. Upon complexation, these transitions underwent slight shifts, confirming ligand-to-metal interaction. For the CuL complex, bands at 257 nm (π→π*), 341 nm (n→π*), and 452 nm were observed. The higher wavelength band (452 nm; 22,123 cm⁻¹) is characteristic of a d–d transition in a square planar geometry, commonly seen in d⁹ Cu(II) complexes. The magnetic moment value of 1.88 BM supports this assignment, as it falls within the expected range for a mononuclear square planar Cu(II) complex with one unpaired electron and slight orbital contribution^23^.

The NiL complex displayed transitions at 267 nm, 339 nm, and 401 nm. The band at 401 nm (24,937 cm⁻¹) is attributed to d–d transitions, consistent with Ni(II) in a tetrahedral geometry, supported by a magnetic moment of 1.77 BM, typical of two unpaired electrons in a high-spin d⁸ tetrahedral configuration^24^. For the CoL complex, peaks at 260 nm, 340 nm, and 404 nm are assigned to π→π*, n→π*, and d–d/LMCT (ligand-to-metal charge transfer) transitions, respectively. The unusually low magnetic moment of 0.64 BM for the CoL complex suggests a low-spin configuration of Co(II), likely induced by a strong ligand field. This behavior is consistent with a square planar geometry, as further supported by the observed spectral features^25,26^. Therefore, based on the spectral assignments and magnetic data, the CuL and CoL complexes are proposed to adopt square planar geometries, while the NiL complex likely exhibits a tetrahedral geometry. The observed LMCT bands in the visible region (352–452 nm) provide additional evidence of metal–ligand interactions, further supporting the proposed coordination modes. The observed LMCT and d–d transitions confirmed successful coordination and geometry assignment, consistent with earlier reports on transition metal schiff base complexes^27,28^.

The molar conductance of the ligand and its CuL, NiL and CoL complexes in DMSO (10^–3^ M) were found to be 0, 10, 8, and 12 Ohm^–1^cm^2^mol^–1^, respectively. By comparing these values with those reported for various complex ions and salts with similar concentrations, it was determined that the complexes are intrinsically non-electrolytic, as indicated by their molar conductivity values (8–15 Ohm^–1^cm^2^mol^–1^)^29^.

The magnetic moment values of CuL, NiL and CoL complexes, presented in Table 1, were measured to be 1.88, 1.77 and 0.64 BM (Bohr Magnetons), respectively. These values along with the spectral data suggest that CuL and CoL complexes have square planner geometries while NiL complex exhibits a tetrahedral structure. Similar spectral and magnetic properties indicating square planar geometry for Cu(II) complexes have been reported by *Shumi et al.*, reinforcing our structural assignment^30^.

**Table 1:** Magnetic moments and electronic spectral data of synthesized ligand and metal complexes.

Compounds	Wavelength(nm)	Wave number(cm^−1^)	Magnetic momentum (Bohr Magneton, BM)	Assignment
L	270 348	37,037 28,735	0	π→π* n→π*
CuL	257 341 452	38,910 29,325 22,123	1.88	π→π* n→π* C.T (L→M)/d-d(T)
NiL	267 339 401	37,453 29,498 24,937	1.77	π→π* n→π* C.T (L→M)/d-d(T)
CoL	260 340 404	38,461 29,411 24,752	0.64	π→π* n→π* C.T(L→M)/d-d(T)

### FT-IR spectra

The FT-IR (Fourier Transform Infrared Spectroscopy) spectrum data of the ligand and its metal complexes are tabulated in Table 2. The analysis revealed significant changes in the stretching frequencies indicating coordination between the ligand and metal ions. The absorption bands at 3434 cm^−1^, 3196 cm^−1^, and 3025 cm^−1^, signifies the ʋ(-OH), ʋ(-NH), and ʋ(-CH) groups, correspondingly. Peaks at 1566 cm⁻¹ and 1692 cm⁻¹ can be attributed to the vibrational modes of the ʋ(C = N) and ʋ(C = O) functional groups^31^.

**Table 2:** FT-IR spectrum data of synthesized ligand and metal complexes.

Compounds	ʋ(-OH)/ cm^−1^	ʋ(C = *N*)/ cm^−1^	ʋ(C = O)/ cm^−1^	ʋ(C-O)/ cm^−1^	ʋ(M-O)/ cm^−1^	ʋ(M-*N*)/ cm^−1^
L	3435	1556	1692	-	-	-
CuL	3434	1501	-	1354	704	657
NiL	3412	1520	-	1372	703	656
CoL	3430	1517	-	1383	702	657

**Fig. 2:**
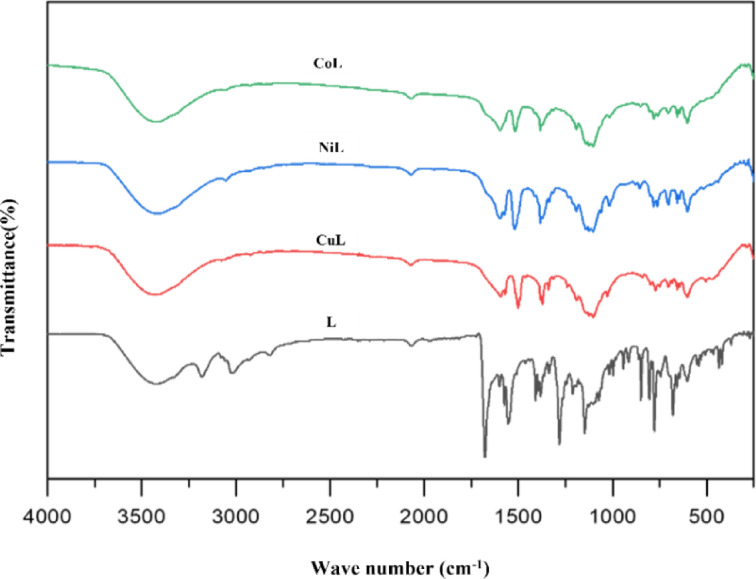
FTIR spectrum of synthesized ligand and metal complexes.

FT-IR spectra of CuL, NiL and CoL complex exhibited azomethine band shift of ligand from 1563 cm⁻¹ to 1501 cm⁻¹, 1520 cm⁻¹ and 1517 cm⁻¹, respectively, indicating metal ion binding to N-atoms^32^. These spectral shifts upon complexation are consistent with reports on schiff base metal complexes, where azomethine and carbonyl bands typically undergo significant changes due to metal coordination^33^. The ʋ(C = O) stretching frequencies were shifted from 1692 cm to 1 to 1354 cm⁻¹, 1372 cm⁻¹ and 1383 cm⁻¹ for CuL, NiL and CoL, respectively, demonstrating influence of oxygen (O) on interactions. Additionally, the identification of novel bond stretching frequencies at approximately 703 cm⁻¹ and 657 cm⁻¹ in the complexes provides evidence of schiff base complexation with metal ions. These characteristic band shifts upon metal coordination, particularly in the azomethine and carbonyl regions, are consistent with earlier FT-IR studies of Schiff base transition metal complexes^27^. Furthermore, the pronounced peak observed in the IR of CuL, NiL and CoL complexes between 3412 and 3434 cm⁻¹ is probably due to the presence of moisture in the KBr particle (Fig. 2).

### ESI-MS spectra

**Fig. 3:**
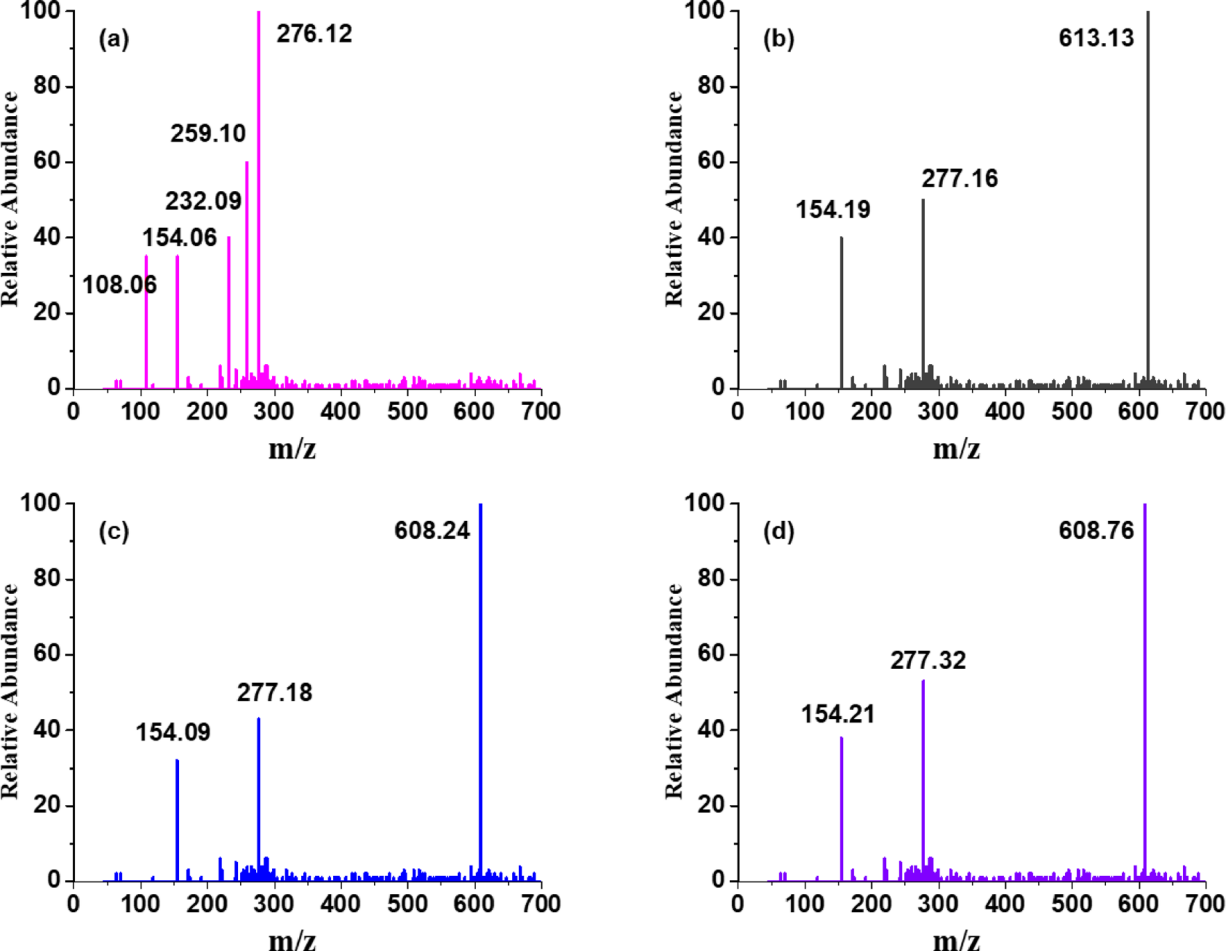
ESI-MS spectra of synthesized **(a)** schiff base ligand (L) and its metal complexes **(b)** CuL (Copper complex), **(c)** NiL (Nickel complex), **(d)** CoL (Cobalt complex).

The ESI-MS spectra of the ligand and its corresponding metal complexes (CuL, NiL, and CoL) were recorded in positive ion mode and are presented in Fig. 3. The ligand spectrum (Fig. 3a) exhibits a base peak at *m/z* 276.12, corresponding to the protonated molecular ion [M + H]⁺, which confirms its molecular weight. Key fragment ions at *m/z* 259.10 (loss of NH_3_), 232.09 (loss of CONH), 154.06 (cleavage near the imine yielding a naphthaldehyde ion), and 108.06 (pyridine-carbonyl ion from amide bond cleavage) support the structural composition of the ligand, including the hydrazide, naphthyl, and pyridyl functionalities. In case CoL complex (Fig. 2b), the major ion peak at *m/z* 613.13 corresponds to [M + H]⁺, confirming the intact molecular ion of the complex. Such molecular ion confirmation and fragmentation patterns have also been reported for Schiff base transition metal complexes^34^. A significant signal at *m/z* 277.16 represents the monoligand [L + H]⁺, while a fragment at *m/z* 154.19 indicates the presence of the isonicotinoylhydrazide unit. Similarly, the NiL complex (Fig. 3c) shows a molecular ion at *m/z* 608.24, consistent with [M + H]⁺, along with fragment peaks at *m/z* 277.18 and 154.09, indicating ligand dissociation and retention of key moieties. The CoL complex (Fig. 3d) displays an analogous fragmentation pattern, with a molecular ion at *m/z* 608.76, and corresponding ligand and sub-ligand fragments at *m/z* 277.32 and 154.21, respectively. The observed fragmentation patterns across all spectra support the proposed structures, affirming the coordination of bidentate Schiff base ligands to the metal centers. The consistency of molecular ion and diagnostic fragment peaks across the metal complexes confirms their structural integrity, the stability of the metal-ligand framework, and successful complex formation.

### Antibacterial activity

**Fig. 4:**
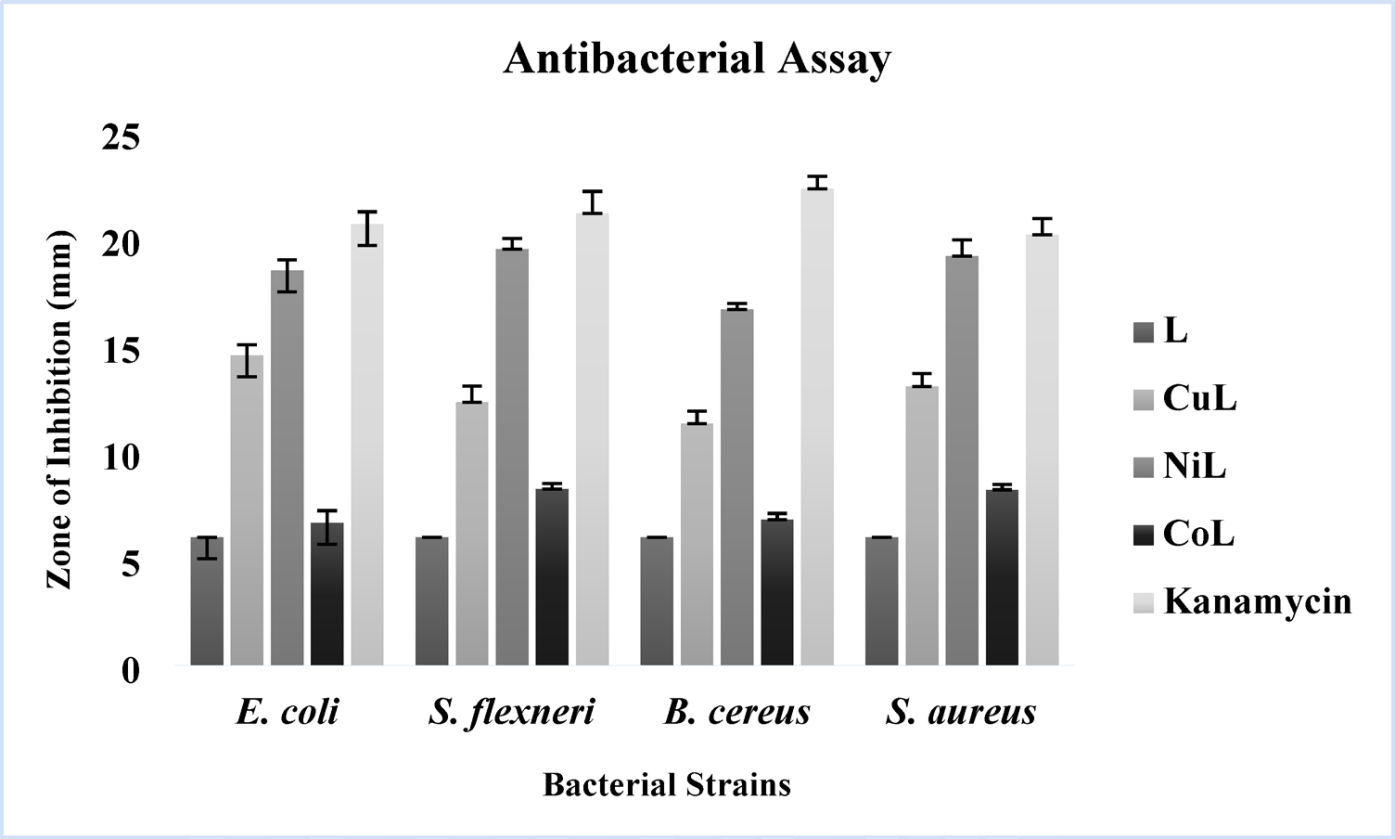
Graphical representation of the antibacterial activity of synthesized compounds.

The synthesized schiff base ligand and its CuL, NiL and CoL complexes were tested for antibacterial activity *in vitro* against two gram-positive bacteria *Staphylococcus aureus* and *Bacillus cereus*, as well as two gram-negative bacteria *Escherichia coli* and *Shigella flexneri*. The observed data were compared to the standard drug Kanamycin as depicted in Table 3 and representative graph is shown in Fig. 4.

**Table 3:** Antibacterial activity of synthesized ligand and metal complexes with kanamycin as control.

Compound	E. coli (mm)	S. flexneri (mm)	B. cereus (mm)	S. aureus (mm)
L	6 ± 0.00	6 ± 0.00	6 ± 0.00	6 ± 0.00
CuL	14.53 ± 5.00	12.33 ± 0.76	11.33 ± 0.58	13.60 ± 0.60
NiL	18.5 ± 0.50	19.5 ± 0.50	16.67 ± 0.29	19.17 ± 0.76
CoL	6.67 ± 0.58	8.27 ± 0.25	6.83 ± 0.29	8.2 ± 0.25
Kanamycin	20.67 ± 0.58	21.17 ± 1.04	22.33 ± 0.58	20.17 ± 0.76

Synthesized ligand and its metal complexes exposed diverse ranges of antibacterial activity with zone of inhibition ranging from 6.67 ± 0.58 mm to 19.5 ± 0.50 mm at the concentration of 50 µg/disc. The free ligand exhibited no significant antibacterial activity. In contrast, the NiL complex demonstrated the highest antibacterial activity, with inhibition zones of 19.5 ± 0.50 mm (*S. flexneri*), 18.5 ± 0.50 mm (*E. coli*), 19.17 ± 0.76 mm (*S. aureus*), and 16.67 ± 0.29 mm (*B. cereus*). The strong antimicrobial activity of Ni–schiff base complexes has also been reported in previous studies against various bacterial pathogens^35,36^. The CuL complex also showed substantial activity, particularly against *E. coli* (14.53 ± 5.00 mm) and *S. aureus* (13.60 ± 0.60 mm). CoL complex exhibited moderate activity, with inhibition zones ranging from 6.67 ± 0.58 to 8.27 ± 0.25 mm.

The poor antibacterial activity of the free ligand could be due to limited lipophilicity and poor cell membrane permeability, which restrict its interaction with bacterial components. In contrast, transition metal coordination with schiff base ligand markedly enhanced biological activity. Similar enhancement of biological function upon complexation has been confirmed in recent reports involving bidentate schiff base ligands^37^. Metal complexation improves the ligand’s solubility and structural stability, both essential for effective function^38^. The resulting metal complexes also adopt defined geometries, such as square planar or tetrahedral, which promote stronger and more specific interactions with bacterial enzymes and cellular components—advantages the free ligand does not possess^38^. Ni is typically known to form stable octahedral structures through coordination with imine nitrogen and phenolic oxygen which increases lipophilicity and improves membrane permeability^35^. Additionally, metal complexes can generate reactive oxygen species (ROS), which damage bacterial components and enhance bactericidal effects. The increased lipophilicity resulting from chelation allows better penetration through bacterial membranes, improving cellular uptake. The overtone concept and chelation theory further explain this behavior: chelation reduces the polarity of the metal ion and increases the delocalization of π-electrons over the chelate ring, enhancing lipophilicity, membrane permeability, and ultimately biological activity^39^. However, the antimicrobial performance of these complexes can also be influenced by other physicochemical factors, including complex geometry, dipole moment, bond lengths, coordination sites, and hydrophobicity^40^. Environmental conditions such as bacterial strain concentration, media composition, incubation time, and diffusion characteristics also affect the observed inhibition zones^41^. The disc diffusion assay offered valuable preliminary insights, and the promising results lay the foundation for further evaluation through MIC determinations, time-kill studies, and in vivo infection models to comprehensively establish the therapeutic potential of these metal complexes.

### *In vitro* antioxidant studies

**Fig. 5:**
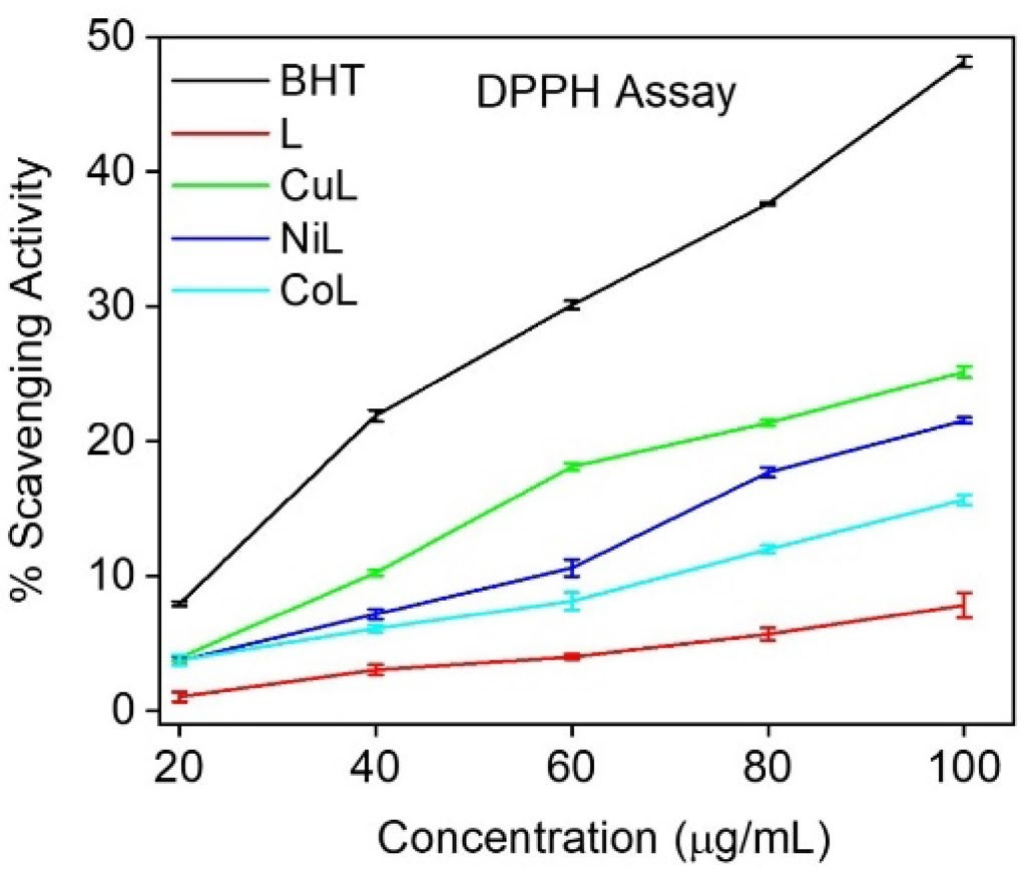
Graphical representation of the antioxidant activity synthesized ligand and metal complexes.

Antioxidants are known to be effective at scavenging DPPH radicals due to their ability to donate hydrogen atoms. Such activities are critical for preventing free radicals from causing harm in a variety of diseases, including AMR. The IC_50_ values of the synthesized compounds and BHT are shown in Table 4 and the representative graph is shown in Fig. 5.

**Table 4:** Antioxidant activity of synthesized ligand and metal complexes with BHT as standard. Data are presented as mean ± SE with three biological replications. Different superscript letters (a–e) indicate statistically significant differences at *p* < 0.05 at Duncan’s multiple range test. These analyses were performed using SAS software (version 9.1.3).

Compound	IC_50_ (µg/ml)
BHT	140.56 ± 0.72^a^
L	673.67 ± 26.60^e^
CuL	187.81 ± 1.42^b^
CoL	339.01 ± 4.13^d^
NiL	224.40 ± 0.95^c^

It can be observed from the results that all the compounds exhibited moderate activity compared to the standard BHT at concentration 20–100 µg/ml. Additionally, all compounds showed statistically significance at *p* < 0.05 compared to the standard BHT (Table 4). The metal complexes exhibited several fold higher DPPH scavenging abilities than the free ligand. Among the complexes, CuL displayed the most potent antioxidant activity with an IC_50_ of 187.81 ± 1.42^b^ µg/ml, followed by NiL (224.40 ± 0.95^c^ µg/ml) and CoL (339.01 ± 4.13^d^ µg/ml), while the free ligand showed the least activity (IC_50_ = 673.67 ± 26.60^e^ µg/ml). This order of activity (CuL > NiL > CoL > L) corresponds well with the redox potential and electronic configuration of the respective metal ions.

The enhanced scavenging effect of the complexes is attributed to the synergistic interaction between the transition metal ions and the schiff base ligand. The variable oxidation states of the metal centers facilitate redox cycling, while the azomethine group of the ligand promotes electron donation, collectively contributing to radical neutralization. Our findings suggest that metal complexation significantly enhances the antioxidant potential of schiff bases. Among the metal complexes, the higher activity of CuL indicates that chelation to the Cu(II) ion further boosts the radical scavenging ability, consistent with previous reports^42^. Despite similar coordination geometry, CoL complex showed weaker antioxidant activity which is likely due to its less favorable redox properties and lower efficiency in electron transfer during radical scavenging. Overall, the coordination between the schiff base ligand and metal ions substantially enhances antioxidant capability, making these complexes promising candidates for mitigating oxidative stress-related damage in biological systems. Additionally, the promising in vitro results provide a strong basis for future in vivo investigations to further validate the antioxidant potential and therapeutic applicability of these metal complexes.

### ADMET analysis

The ADMET assessments are crucial for computer aided drug development as they help predict adverse effects and guide the structural modifications needed to enhance safety profiles. Such analyses are fundamental in preclinical studies to mitigate health risks and ensuring patient safety and therapeutic efficacy. The ADMET analysis of schiff base ligand and its metal complexes using SwissADME, pkCSM and Protox-3 revealed significant findings regarding their drug-likeness and toxicity profiles.

SwissADME evaluates the drug likeness properties on the basis of Lipinski’s and Veber’s rules. The Lipinski’s Rule of Five predicts oral bioavailability based on four criteria: molecular weight ≤ 500, logP ≤ 5, ≤5 hydrogen bond donors, and ≤ 10 hydrogen bond acceptors. Compounds meeting at least three are considered drug-like^43^. Veber’s Rule suggests that a compound should have ≤ 10 rotatable bonds and a topological polar surface area (TPSA) ≤ 140 Å² for optimal oral bioavailability^43^.

As shown in Table 5, the ligand strictly adhered to Lipinski’s Rule of Five, exhibiting a molecular weight (MW) below 500 g/mol, acceptable logP (2.18), limited hydrogen bond donors and acceptors. In contrast, the metal complexes exceeded the MW threshold, accounting for one Lipinski violation in each. Nevertheless, they retained acceptable logP values and favorable topological polar surface areas (TPSA), complying with Veber’s criteria. Rotatable bond counts were within limits, ensuring sufficient molecular flexibility for bioavailability. The bioavailability score remained identical (0.55) across all compounds, suggesting the retention of oral absorption potential despite increased molecular mass.

**Table 5:** *In Silico* prediction of drug-likeness properties (by SwissADME).

		Compound Name			
		**L**	**CuL**	**NiL**	**CoL**
Lipinski Rules	MW (g/mol) < 500	275.30	612.14	607.29	541.38
	HBA < 10	3	8	8	8
	HBD < 5	1	0	0	0
	Log_P_ (Mlog_p_) < 5	2.18	4.06	4.06	4.06
	Lipinski violation	0	1	1	1
Veber’s Rules	nRB ≤ 10	4	10	10	10
	TPSA (Å²) ≤ 140	54.35	93.68	93.68	93.68
Physicochemical Properties	Num. of heavy atom	21	43	37	37
	Molecular refractivity	82.91	166.80	166.80	166.80
	Bioavailability	0.55	0.55	0.55	0.55

Pharmacokinetic parameters from Table 6 showed that the free ligand exhibited high gastrointestinal absorption and blood-brain barrier (BBB) permeability, along with high CaCO_2_ permeability, indicating strong systemic and central nervous system (CNS) accessibility. Despite a slight decrease in CaCO_2_ permeability, the metal complexes maintained excellent human intestinal absorption. However, they exhibited lower BBB permeability, which, while reducing CNS (Central nervous System) access, is often beneficial for minimizing neurological side effects in non-CNS-targeting drugs. The limited central nervous system accessibility of the metal complexes also ensures prevention of potential side effects in systemic treatments^44^. Notably, only the metal complexes were predicted to inhibit both P-glycoprotein I and II, suggesting potential alterations in cellular efflux and distribution. Additionally, the fraction unbound (Fu) values were significantly higher in metal complexes than in the ligand, indicating improved systemic distribution and reduced plasma protein binding upon metal coordination. In terms of metabolism, all compounds were predicted as CYP3A4 substrates and CYP2C19 inhibitors. The ligand showed additional inhibition of CYP1A2, while the metal complexes uniquely inhibited CYP2C9. These findings suggest that metal coordination can influence the metabolic fate of the ligand, which may impact drug-drug interaction potential. Furthermore, all complexes displayed higher systemic clearance compared to the ligand, indicating an enhanced excretion profile, possibly reducing systemic accumulation and toxicity.

**Table 6:** *In Silico* prediction of ADMET properties (by Pkcsm).

	Parameter	Ligand	CuL	NiL	CoL
Absorption	Water Solubility (log mol/L)	–3.422	–4.482	–4.483	–4.482
	CaCO_2_ Permeability (log Papp (10⁻⁶ cm/s))	1.382	1.133	1.132	1.132
	Intestinal Absorption (% Absorbed)	94.7	100	100	100
	Skin Permeability (log K_p_ (cm/s))	–2.302	–2.735	–2.735	–2.735
	P-glycoprotein I & II Inhibition	No	Yes	Yes	Yes
Distribution	BBB Permeability (log BB)	0.351	–1.243	–1.242	–1.243
	CNS Permeability (log PS)	–2.049	–1.886	–1.886	–1.886
	Fraction Unbound (Fraction (Fu))	0.074	0.22	0.22	0.22
Metabolism	CYP3A4 Substrate	Yes	Yes	Yes	Yes
	CYP1A2 Inhibition	Yes	No	No	No
	CYP2C19 Inhibition	Yes	Yes	Yes	Yes
	CYP2C9 Inhibition	No	Yes	Yes	Yes
Excretion	Total Clearance log mL/min/kg	0.73	3.257	3.249	3.250
Toxicity	AMES Toxicity	Yes	No	No	No
	Hepatotoxicity	Yes	No	No	No
	hERG II Inhibition	No	Yes	Yes	Yes
	Max Tolerated Dose (log mg/kg/day)	0.07	0.62	0.621	0.62
	Acute Oral Toxicity (Rat LD₅₀) (mol/kg)	2.152	2.941	2.941	2.941
	Chronic Toxicity (LOAEL) (log mg/kg/day)	1.85	0.146	0.146	0.146

Toxicological evaluations from Tables 6 and 7 presented an overall safer profile for the metal complexes. While the ligand showed probable carcinogenicity and tested positive for AMES toxicity and hepatotoxicity, all metal complexes were negative for these toxicities, indicating a reduced risk profile post-coordination. Predicted LD_50_ values further support this trend: the ligand had a higher LD_50_, while metal complexes showed significantly lower values, implying greater potency and reduced acute toxicity.

**Table 7:** *In Silico* toxicity profiling of drug-likeliness properties (Protox-3).

Chemical identifier	Types of toxicity					
	Cytotoxicity (probability)	Nephrotoxicity(probability)	Immunogenicity(probability)	Carcinogenicity (probability)	Mutagenicity (probability)	Predicted LD_50_ mg/kg
L	0.82(inactive)	0.61(inactive)	0.86(inactive)	0.74(active)	0.68(inactive)	710
CuL	0.80(inactive)	0.61(inactive)	0.95(inactive)	0.54(inactive)	0.67(inactive)	134
NiL	0.80(inactive)	0.61(inactive)	0.95(inactive)	0.54(inactive)	0.67(inactive)	134
CoL	0.80(inactive)	0.61(inactive)	0.96(inactive)	0.54(inactive)	0.67(inactive)	134

Collectively, the ADMET results suggest that although the metal complexes slightly compromise some drug-likeness parameters, they offer notable advantages in terms of safety, systemic clearance, and metabolic stability. The coordination to metal centers not only enhances pharmacokinetic robustness but also mitigates predicted carcinogenic and hepatotoxic risks. These findings underscore the potential of metal-ligand complexes as improved therapeutic candidates, warranting further in vivo validation.

### Docking studies

**Fig. 6:**
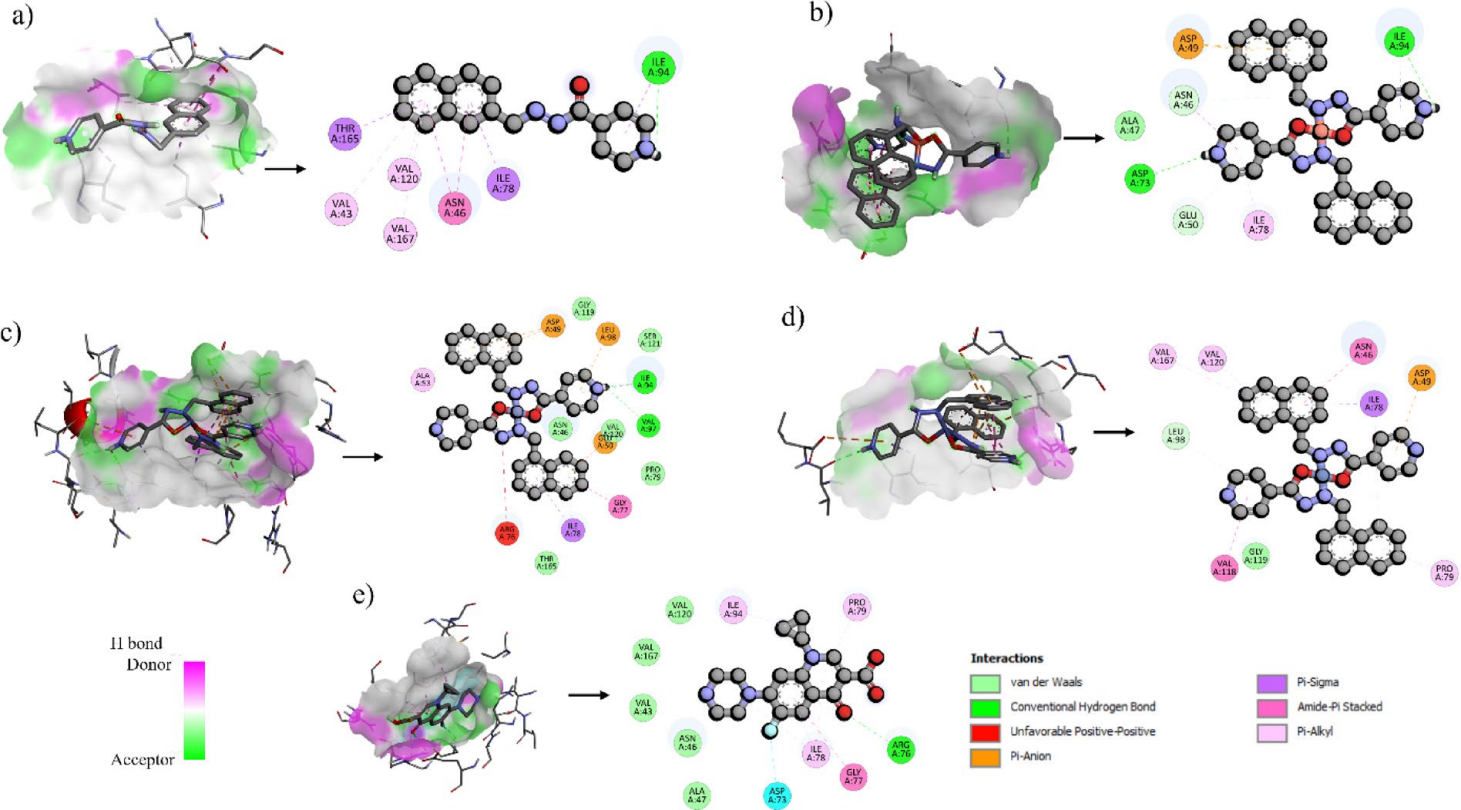
Molecular docking interactions of the synthesized **(a)** schiff base ligand (L) and its metal complexes **(b)** CuL (Copper complex), **(c)** NiL (Nickel complex), **(d)** CoL (Cobalt complex) and **(e)** control (ciprofloxacin) pose, and 2D view.

To validate the experimental study on the biological activities of the free ligand and their metal complexes, we have chosen an appropriate protein (DNA Gyrase; PDB ID: 7P2M) and docked the complexes against them. Ciprofloxacin was used as the standard drug. The binding energies as well as different bonds for protein inhibition are presented in Table 8 and the varied interactions formed with the target protein is shown in Fig. 6.

**Table 8:** Molecular docking interaction of synthesized ligand and metal complexes against DNA gyrase (PDB ID: 7P2M).

Complex	Binding energy (kcal/mol)	Amino acid residues	Bond types
L + 7P2M	−8	A: ILE94 A: ILE78 A: ILE94 A: THR165 A: ASN46 A: ASN46 A: VAL43 A: VAL120 A: VAL167	Hydrogen Bond Pi-Sigma Pi-Sigma Pi-Sigma Amide-Pi Stacked Amide-Pi Stacked Pi-Alkyl Pi-Alkyl Pi-Alkyl
CuL + 7P2M	−8	A: ILE94 A: ASP73 A: ASN46 A: GLU50 A: ASP49 A: ASP49 A: ILE94 A: ASN46 A: ILE78	Hydrogen Bond Hydrogen Bond Carbon Hydrogen Bond Carbon Hydrogen Bond Pi-Anion Pi-Anion Pi-Sigma Amide-Pi Stacked Pi-Alkyl
CoL + 7P2M	−8.7	A: ILE94 A: VAL97 A: ASP49 A: ASP49 A: GLU50 A: LEU98 A: ILE78 A: ILE94 A: GLY77 A: ALA53 A: ILE78	Hydrogen Bond Hydrogen Bond Pi-Anion Pi-Anion Pi-Anion Pi-Anion Pi-Sigma Pi-Sigma Amide-Pi Stacked Pi-Alkyl Pi-Alkyl
NiL + 7P2M	−9.9	A: LEU98 A: ASP49 A: ILE78 A: ASN46 A: VAL118 A: VAL120 A: VAL167 A: ILE78 A: PRO79	Carbon Hydrogen Bond Pi-Anion Pi-Sigma Amide-Pi Stacked Amide-Pi Stacked Pi-Alkyl Pi-Alkyl Pi-Alkyl Pi-Alkyl
Control + 7P2M	−7.3	A: ARG76 A: ARG76 A: ASP73 A: GLY77 A: ILE78 A: ILE94 A: ILE78 A: PRO79 A: ILE78	Hydrogen Bond Hydrogen Bond Halogen Amide-Pi Stacked Alkyl Alkyl Pi-Alkyl Pi-Alkyl Pi-Alkyl

Among the tested compounds, NiL exhibited the most favorable binding energy (−9.9 kcal/mol), outperforming CoL (−8.7 kcal/mol), CuL and the free ligand L (both − 8.0 kcal/mol), and the control (−7.3 kcal/mol). These results indicate that all metal complexes interacted more strongly with DNA Gyrase, suggesting improved inhibitory potential, consistent with earlier findings where hydrazone-based transition metal complexes displayed strong docking interactions with microbial enzymes^45^. In a previous study, schiff base derivatives of ciprofloxacin showed docking scores of − 8.2 and − 8.5 kcal/mol against bacterial DNA gyrase, supporting their role as effective inhibitors^46^. Likewise, a nickel complex demonstrated a docking score of approximately − 8.2 kcal/mol against DNA gyrase B of *Staphylococcus aureus*, indicating a strong interaction with the active site^47^. Compared to these, the NiL complex in the present study exhibited superior binding affinity (–9.9 kcal/mol), further validating its therapeutic promise.

The diverse interactions of NiL shown in Fig. 6d—including carbon hydrogen bonds, pi-anion, pi-sigma, and extensive pi-pi stacking—highlight its strong binding, better occupancy, and specificity within the active site. The electronic structure of Ni(II) allows for significant π-backbonding, where electron density is transferred from filled metal d-orbitals to empty π* orbitals of the ligands. This interaction stabilizes the complex and enhances binding affinity to proteins^48^. CoL formed two conventional hydrogen bonds, along with pi-anion and pi-cation interactions (Fig. 6c), contributing to electrostatic stability. CuL, despite sharing the same binding energy as L, displayed a more varied interaction profile, including hydrogen bonds, carbon-hydrogen bonds, pi-anion, and multiple pi-pi stacking interactions (Fig. 6b).

These findings emphasize the importance of metal coordination in drug design. Cu(II), Co(II), and Ni(II) enhance protein binding through their flexible coordination geometries and strong affinity for nitrogen and oxygen donor atoms. Cu(II) typically adopts square planar geometry, favoring π-type interactions. The high-affinity binding often involves histidine and other amino acid residues, with the structural flexibility and surface accessibility of these sites being crucial for binding strength and specificity^49^. Co(II) and Ni(II) often exhibit octahedral geometries, enabling stable and specific coordination within protein active sites. Their redox properties and electronic configurations further support strong and adaptable binding. Additionally, our docking scores are comparable to those reported for schiff base and hydrazone metal complexes, confirming that our synthesized complexes exhibit similar or stronger binding affinities, supporting their therapeutic relevance^50,51^.

### Molecular dynamic simulation studies

**Fig. 7:**
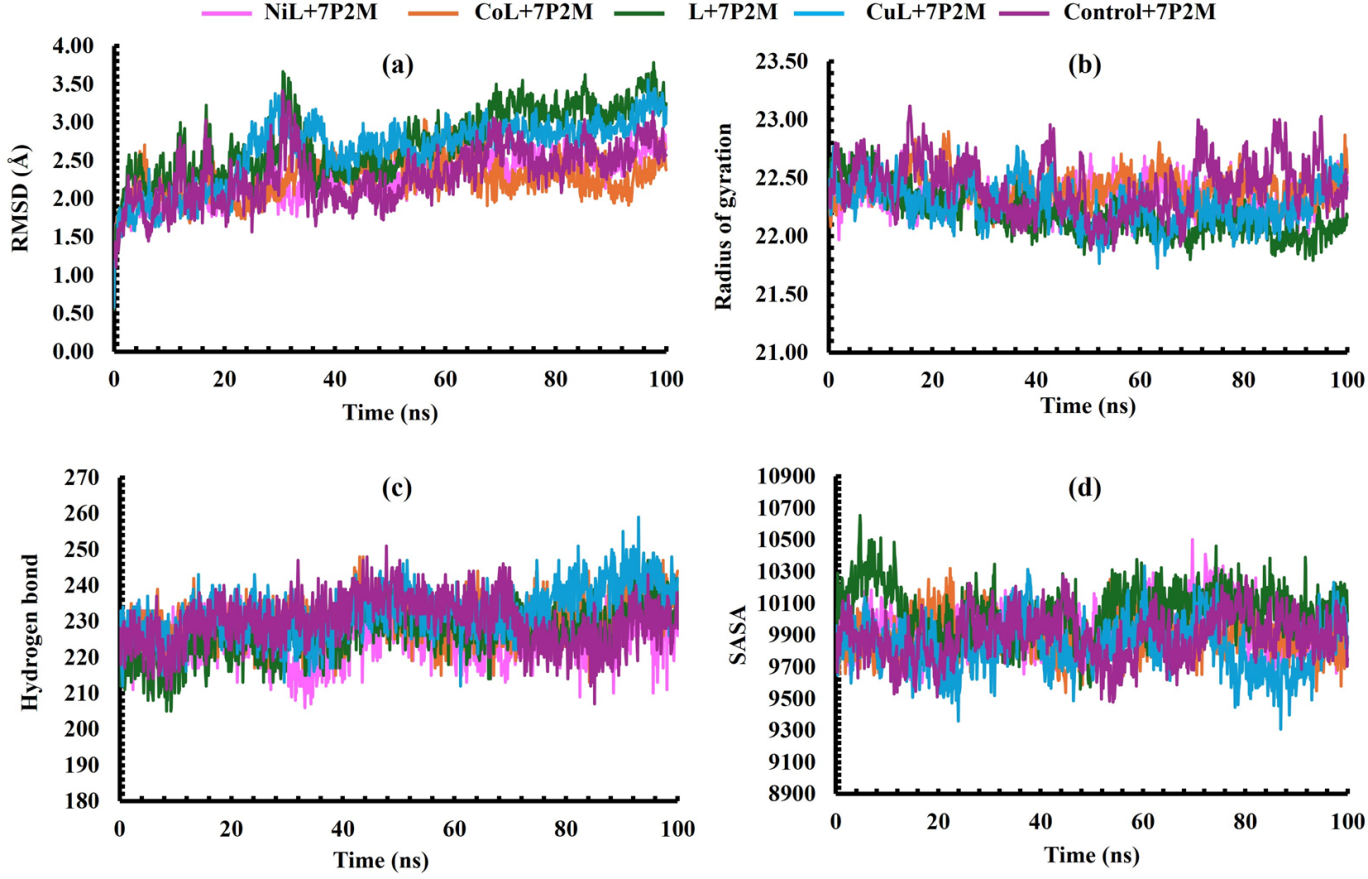
MD simulation of DNA Gyrase (PDB ID: 7P2M) complexes with the synthesized ligand and its metal-ligand complexes. (**a**) RMSD, (**b**) radius of gyration, (**c**) hydrogen bond, and (**d**) SASA at 100 ns simulation period.

Molecular dynamics (MD) simulations were performed to further validate the docking outcomes and assess the stability of DNA Gyrase (PDB ID: 7P2M) complexes with the synthesized ligand and its metal-ligand complexes in comparison with the control system (Fig. 7). The simulations provided insights into the conformational stability, compactness, hydrogen bonding, and solvent exposure of the complexes under physiological conditions. Here, we analyzed multiple parameters on the MD trajectories, including Root Mean Square Deviation (RMSD), Root Mean Square Fluctuations (RMSF), Hydrogen Bonds (H-Bond), Radius of Gyration (Rg), and Solvent Accessible Surface Areas (SASA).

RMSD analysis in Fig. 7a revealed that NiL + 7P2M and CoL + 7P2M displayed stable trajectories with identical average RMSD values of 2.25 Å, similar to the control (2.30 Å), indicating minimal backbone perturbations. By contrast, L + 7P2M showed the highest deviation (2.74 Å), suggesting reduced stability, while CuL + 7P2M exhibited moderate fluctuations with an RMSD of 2.62 Å. For small globular proteins, RMSD fluctuations within 1–3 Å are generally acceptable, whereas values beyond this range indicate significant conformational changes^52^. Since the candidate complexes demonstrated stable behavior through average RMSD values, it can be said that the enzyme structure remained consistent during the simulations. The results further confirmed that the ligand and metal complexes stayed securely within the active site without noticeable diffusion or detachment from their original binding position.

The Rg measures the overall compactness of a protein or protein-ligand complex during molecular dynamics simulations^53^. In our study, all complexes exhibited stable Rg values throughout the simulation, indicating that ligand binding did not significantly destabilize the protein structure (Fig. 7b). The slightly lower Rg observed for L + 7P2M (22.18 Å) suggests that this ligand may induce a marginally more compact protein conformation, potentially reflecting tighter ligand-protein interactions or reduced flexibility in certain regions. Conversely, CoL + 7P2M, with the highest Rg (22.41 Å), may allow slightly more structural fluctuations, though the difference is minimal and unlikely to impact overall stability. These results collectively indicate that all ligand-bound systems maintained structural integrity comparable to the control, supporting the potential of these ligands to form stable complexes without causing major conformational perturbations.

Hydrogen bond analysis is essential for understanding the strength and persistence of protein-ligand interactions during MD simulations. The detection and characterization of hydrogen bonds can significantly influence the interpretation of binding affinities and stability of complexes. In this study, CuL + 7P2M formed the highest average number of hydrogen bonds (232.74), followed closely by CoL + 7P2M (230.53) and the control system (229.95) shown in Fig. 7c. The elevated hydrogen bond counts in these complexes suggest that both copper and cobalt derivatives established strong, stable, and dynamic interactions with the target protein, reinforcing their potential as robust binders. On the other hand, NiL + 7P2M and L + 7P2M exhibited comparatively fewer hydrogen bonds (224.49 and 226.91, respectively), which may account for their lower stability and higher fluctuations observed in other analyses (e.g., RMSD and SASA).

SASA analysis provided further insight into solvent exposure and structural dynamics of the complexes. Among the systems, L + 7P2M showed the highest SASA value (10,053.37 Å²), reflecting greater interaction with the solvent, which may correspond to reduced rigidity and a less stable binding environment (Fig. 7d). In contrast, CuL + 7P2M displayed the lowest SASA (9,829.15 Å²), consistent with a more compact, solvent-shielded structure that favors stronger protein-ligand interactions. The control (9,892.42 Å²), NiL + 7P2M (9,940.81 Å²), and CoL + 7P2M (9,902.19 Å²) showed intermediate values, indicating moderate solvent accessibility and stable conformations.

Collectively, these results suggest that Co + 7P2M and Cu + 7P2M maintained more stable protein-ligand interactions, as reflected by favorable RMSD, hydrogen bond counts, and SASA values, whereas L + 7P2M displayed comparatively reduced stability due to higher RMSD and SASA, despite maintaining compactness in terms of Rg. Thus, the simulations highlight Cu and Co derivatives of 7P2M as more promising candidates, given their ability to preserve structural stability and strong interactions throughout the 100 ns simulation.

### DFT computational studies

#### The geometry of molecules

**Fig. 8:**
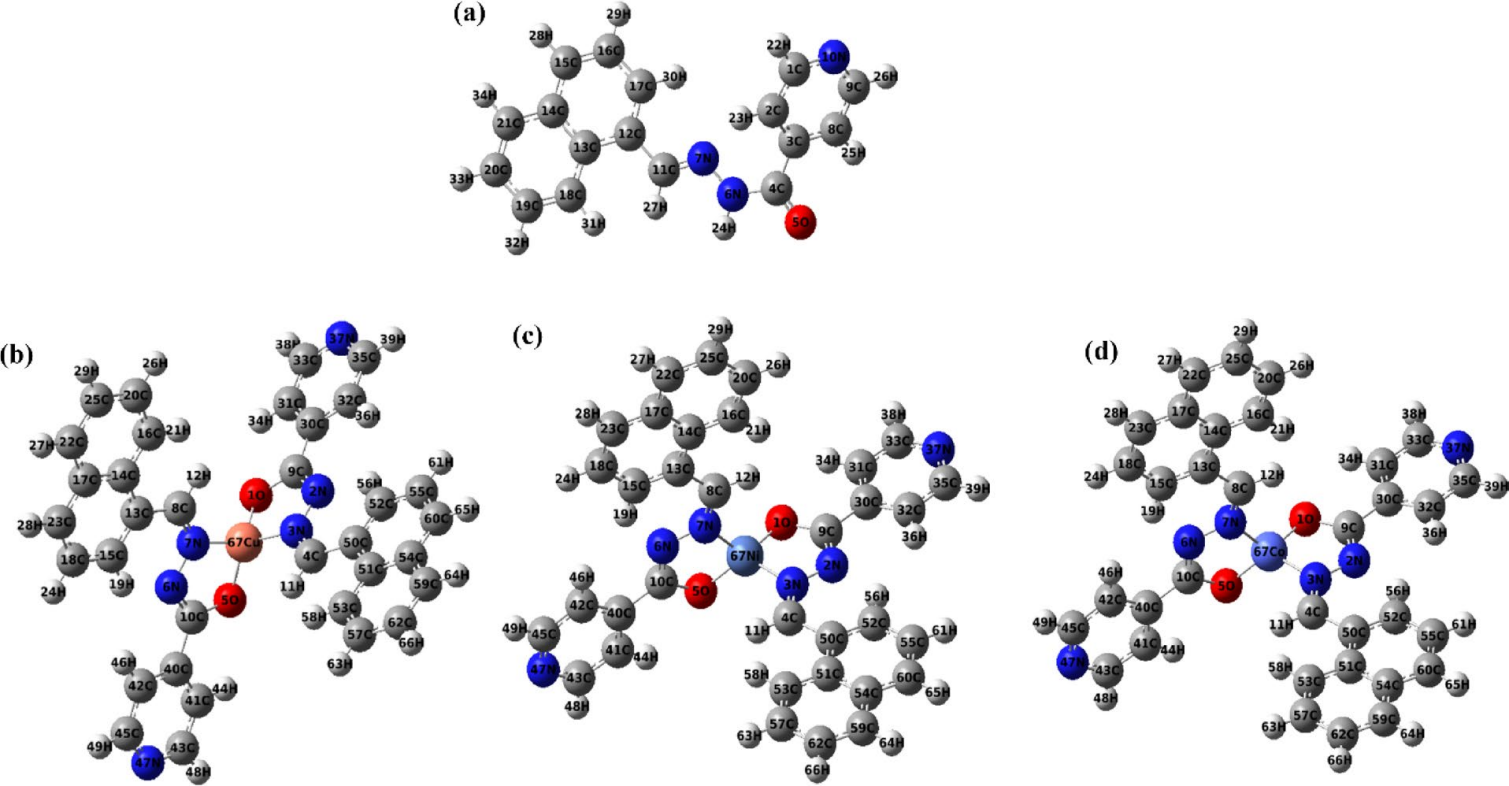
Optimized 3D geometries of **(a)** schiff base ligand (L) and its **(b)** Copper complexes (CuL), **(d)** Nickel complex (NiL) and **(c)** Cobalt complex (CoL).

The geometries of the free ligand and its metal complexes (CuL, NiL, CoL) were optimized using Density Functional Theory (DFT), employing the B3LYP functional in conjunction with the LANL2DZ basis set. The optimized 3D geometries are presented in  Fig. 8. The optimized geometrical parameters of the free ligand confirm its characteristic structural features and coordination potential. The C–C bond lengths within the aromatic rings (Table 9) fall within the typical range for delocalized π-systems which indicates aromatic stabilization^54^. The C4–O5 bond length of 1.220 Å is indicative of a carbonyl (C = O) group, while the C4–N6 (1.382 Å) and N6–N7 (1.357 Å) bond lengths are consistent with the presence of a conjugated imine and azo linkages^55^. The N7–C11 (1.283 Å) and N10–C9 (1.337 Å) bonds further support extended conjugation involving nitrogen atoms. The uniformity of the C–H bond lengths (1.080–1.093 Å) suggests that the ligand is geometrically stable and free from significant steric strain. These values imply effective coordination of the donor atoms (N and O) to metal centers, aligning with trends observed in related coordination systems, and are in line with recent DFT studies on Schiff base metal complexes that highlighted similar stability and electronic properties^56^.

**Table 9:** Geometrical parameters of schiff-base ligand.

Bond connectivity	Bond length Value (Å)	Bond connectivity	Bond length Value (Å)	Bond connectivity	Bond length Value (Å)
C1-C2	1.39371	C13-C14	1.43460	N6-H24	1.01981
C2-C3	1.39648	C14-C15	1.41711	C8-H25	1.08252
C3-C4	1.50127	C15-C16	1.37504	C9-H26	1.08609
C4-O5	1.22030	C16-C17	1.40555	C11-H27	1.09325
C4-N6	1.38201	C13-C18	1.42201	C15-H28	1.08482
N6-N7	1.35785	C18-C19	1.37519	C16-H29	1.08394
N7-C11	1.28311	C19-C20	1.41262	C17-H30	1.08290
N10-C9	1.33724	C20-C21	1.37241	C18-H31	1.08221
C8-C9	1.39118	C21-C14	1.42039	C19-H32	1.08416
C11-C12	1.46612	C1-H22	1.08630	C20-H33	1.08392
C12-C13	1.43769	C2-H23	1.08027	C21-H34	1.08493

The geometrical parameters of the synthesized CuL, NiL, and CoL complexes summarized in Table 10 revealed notable differences in bond lengths and bond angles, reflecting variations in coordination environments and the metal-ligand interactions.

The CuL complex demonstrated shorter metal–ligand bond lengths—Cu–O (1.819–1.820 Å) and Cu–N (1.831–1.834 Å) that suggests stronger metal-ligand interactions and higher structural stability. Bond angles such as N7–Cu–O1 (123.38°), N7–Cu–N3 (114.89°), and O1–Cu–O5 (115.33°) suggest a relatively undistorted square planar geometry, supported by the planar preference of nitrogen and oxygen donors, which typically range around 90° and 180° for equatorial and axial positions, respectively. This stability is further supported by the coordination of nitrogen and oxygen, which tend to favor planar arrangements due to their orbital orientations^57^. In contrast, the NiL complex displayed longer Ni–O (~ 1.856 Å) and Ni–N (~ 1.916 Å) bond lengths, with distorted angles like N7–Ni–O1 (124.00°) and O1–Ni–O5 (91.79°), suggesting a distorted octahedral geometry. Longer bond lengths can lead to the formation of octahedral structures due to the spatial arrangement and steric requirements of the ligands or atoms involved. These distortions indicate angular strain and weaker metal–ligand interactions. CoL complex showed the longest metal–ligand bonds—Co–O (~ 1.965 Å) and Co–N (~ 2.06 Å)—along with significantly distorted angles, like O1–Co–O5 (147.83°) and N7–Co–N3 (143.17°). These features point toward a highly distorted octahedral geometry, influenced by cobalt’s larger ionic radius and reduced bonding strength. The ionic radius of cobalt allows it to accommodate six ligands around it, leading to an octahedral coordination geometry. Moreover, ligand backbone bond lengths (such as, C13–C8, C8–N5, N6–C10, C10–O5) remain largely consistent across all complexes, maintaining the structural integrity of the schiff-base framework. The presence of conjugated bonds such as C = N and N = N in all complexes confirms electron delocalization enhancing stability and reactivity.

**Table 10:** Geometrical parameters of metal complexes.

CuL		NiL		CoL	
Bond length (Å)	**Bond angle (°)**	**Bond length(Å)**	**Bond angle (°)**	**Bond length** **(Å)**	**Bond angle** **(°)**
C13-C8 1.54273	N7-Cu67-O1 123.38229	C13-C8 1.43086	N7-Ni67-O1 124.00270	C13-C8 1.43089	N7-Co67-O1 110.92818
C8-N5 1.29527	N7-Cu67-O5 91.60985	C8-N5 1.35324	N7- Ni 67-O5 82.23771	C8-N5 1.35306	N7-Co67-O5 79.50269
N7-N6 1.39594	N7-Cu67-N3 114.89862	N7-N6 1.37597	N7- Ni 67-N3 107.17841	N7-N6 1.36735	N7-Co67-N3 143.17736
N6-C10 1.32871	N3-Cu67-O1 91.60024	N6-C10 1.36587	N3- Ni 67-O1 82.23817	N6-C10 1.38726	N3-Co67-O1 79.50284
C10-O5 1.46303	O1-Cu67-O5 115.33403	C10-O5 1.31667	O1- Ni 67-O5 91.79183	C10-O5 1.31082	O1-Co67-O5 147.83749
C10-C40 1.53947	Cu67-N7-C8 125.01276	C10-C40 1.46752	Ni 67-N7-C8 124.08308	C10-C40 1.46548	Co67-N7-C8 124.72792
O5-Cu67 1.81945	Cu67-N7-N6 109.59852	O5-Ni67 1.85617	Ni 67-N7-N6 114.25602	O5-Co67 1.96533	Co67-N7-N6 113.80606
N3-Cu67 1.83139	Cu67-O1-C9 104.28803	N3- Ni 67 1.91643	Ni 67-O1-C9 113.26745	N3-Co67 2.06015	Co67-O1-C9 113.79844
O1-Cu67 1.82053	Cu67-O5-C10 104.23908	O1- Ni 67 1.85616	Ni 67-O5-C10 113.26754	O1-Co67 1.96532	Co67-O5-C10 113.79839
N7-Cu67 1.83418	Cu67-N3-N2 109.78729	N7- Ni 67 1.91643	Ni 67-N3-N2 114.25568	N7-Co67 2.06014	Co67-N3-N2 113.80571
C9-N2 1.32753	Cu67-N3-C4 124.87234	C9-N2 1.36586	Ni 67-N3-C4 124.08335	C9-N2 1.38726	Co67-N3-C4 124.72825
N3-N4 1.39473	C4-N3-N2 125.34000	N3-N4 1.35324	C4-N3-N2 120.46442	N3-N4 1.35306	C4-N3-N2 121.17599
C4-C50 1.54062	N3-N2-C9 113.94588	C4-C50 1.43086	N3-N2-C9 109.65782	C4-C50 1.43089	N3-N2-C9 111.31331
C43-N47 1.34343	N2-C9-O1 119.40892	C43-N47 1.35484	N2-C9-O1 119.44920	C43-N47 1.36010	N2-C9-O1 121.53734
N47-C45 1.34340	C8-N7-N6 125.38762	N47-C45 1.35319	C8-N7-N6 120.46450	N47-C45 1.35822	C8-N7-N6 121.17594
C35-N37 1.34394	N7-N6-C10 113.87566	C35-N37 1.35319	N7-N6-C10 109.65776	C35-N37 1.35822	N7-N6-C10 111.31326
N37-C33 1.34385	N6-C10-O5 119.45591	N37-C33 1.35484	N6-C10-O5 119.44889	N37-C33 1.36010	N6-C10-O5 121.53724

#### Investigation of atomic partial charge

**Fig. 9:**
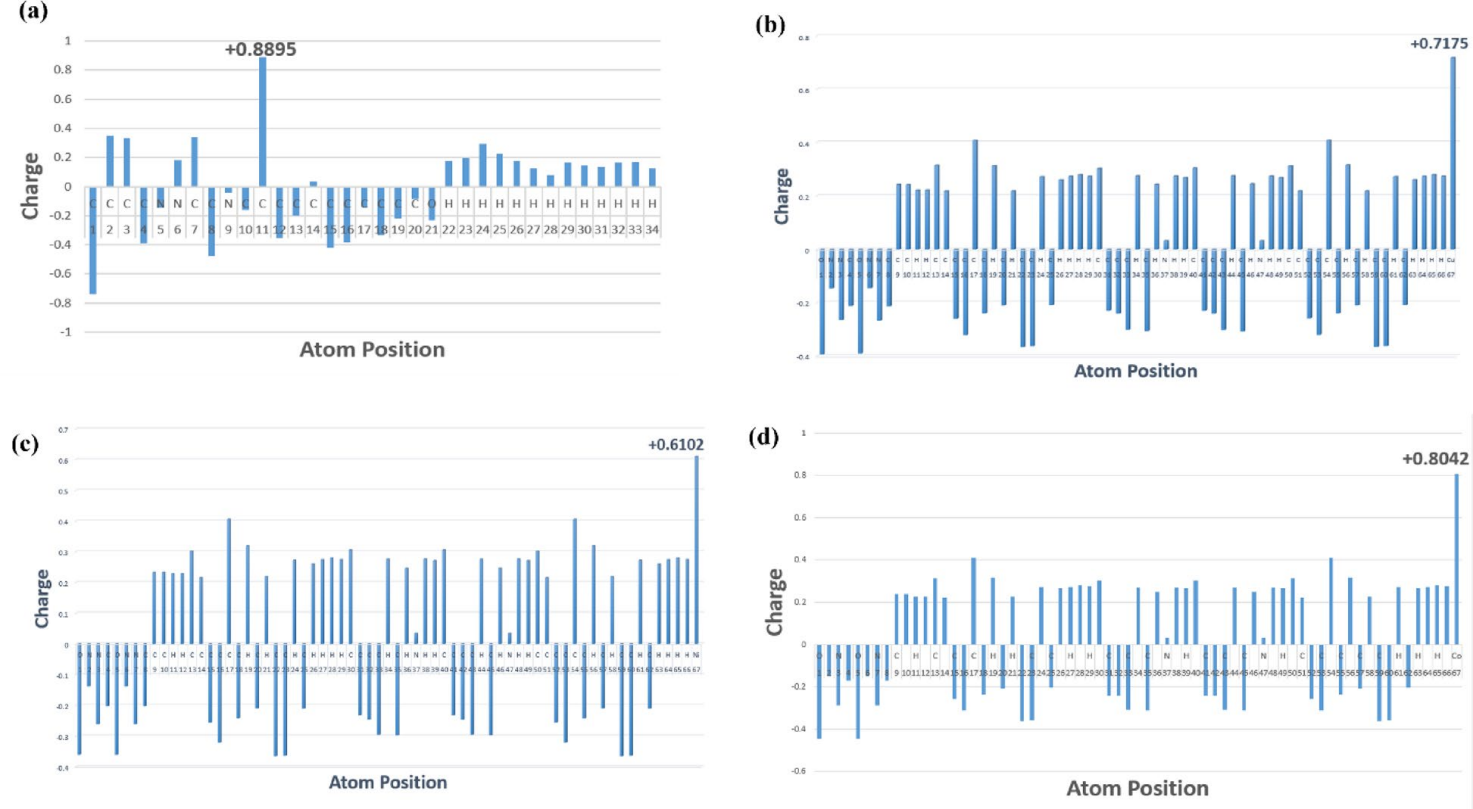
Atomic partial charge plot of **(a)** schiff base ligand (L) and its **(b)** Copper complexes (CuL), **(d)** Nickel complex (NiL) and **(c)** Cobalt complex (CoL).

The atomic partial charges of the ligand and its metal complexes were calculated in the gas phase using the B3LYP/6–311 + + G(d, p) basis set for the ligand and the LANL2DZ basis set for the metal complexes. These calculations offered valuable insights into their electronic structures and vibrational properties. Atomic partial charges reflect the distribution of electron density across a molecule, which is crucial for understanding its chemical behavior, including potential reaction sites and molecular interactions^58^. Figure 9 illustrates the atomic partial charge distribution for both the ligand and its metal complexes in detail.

For the free ligand, notable positive partial charges were seen, especially on specific carbon atoms. In particular, C10 had a charge of + 0.8895, likely due to the influence of nearby electronegative oxygen and nitrogen atoms that withdraw electron density^59^. The imine nitrogen and oxygen atoms also contributed significantly to the overall charge distribution, causing electron deficiency in the adjacent carbon atoms because of their strong electronegativity^60^. In the case of the metal complexes, the metal centers—Cu(II), Ni(II), and Co(II)—showed higher positive partial charges of + 0.7175, + 0.6102, and + 0.8042, respectively. These charges mainly result from coordination with two oxygen and two imine nitrogen atoms from the ligand^61^. Such coordination promotes a more uniform charge distribution, where the metal’s positive charge is moderated by the electron-donating ability of the ligand’s oxygen and nitrogen atoms^32^. Similar findings have been reported where positive atomic charge distribution in schiff base metal complexes was linked to enhanced biological interactions^62^. These variations in atomic charges reflect electron redistribution upon metal coordination, which may enhance electrostatic interactions with microbial enzymes or cell membranes, thereby contributing to improved biological activity.

#### Molecular electrostatic potential (MEP) analysis

**Fig. 10:**
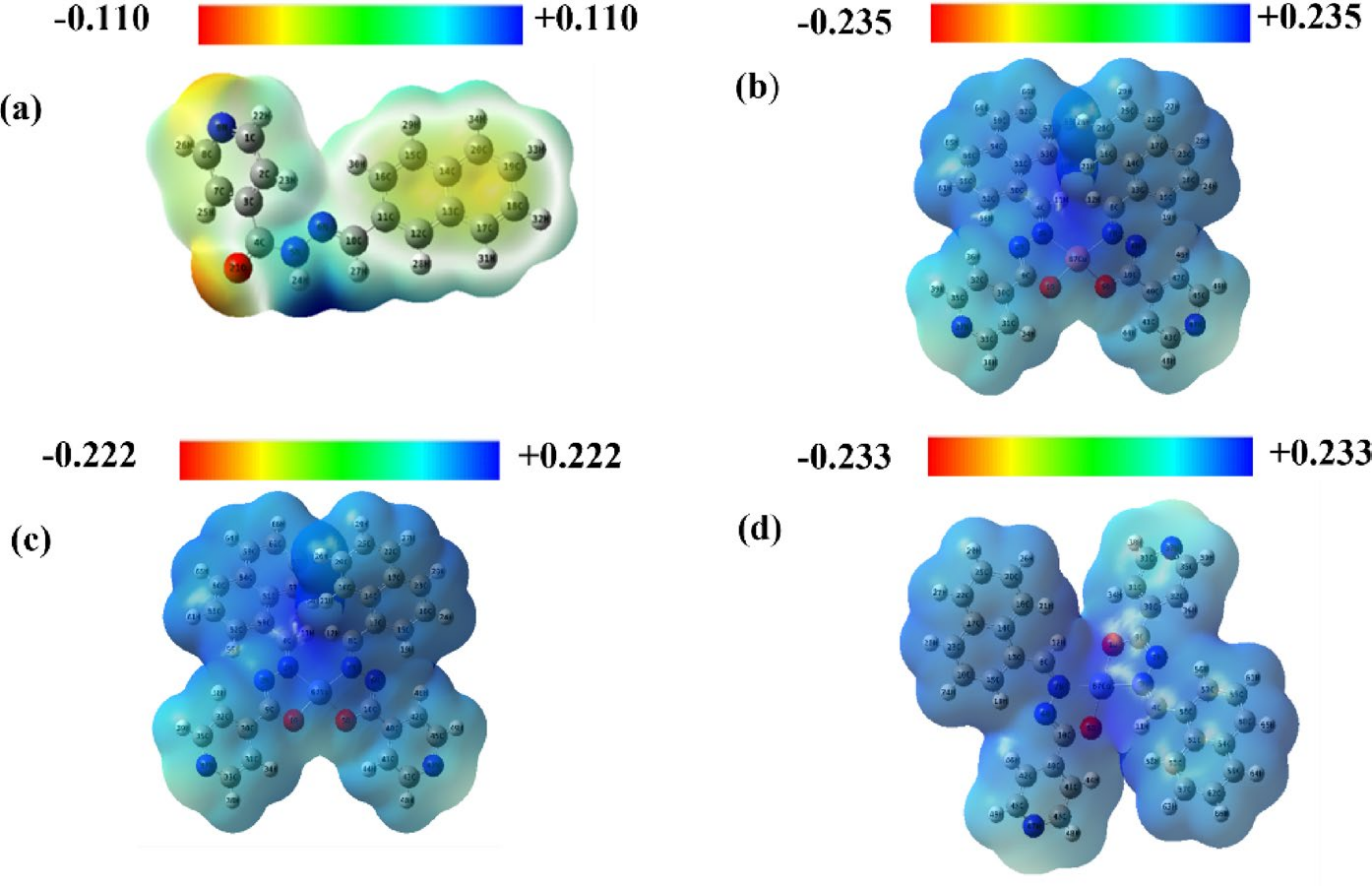
Molecular electrostatic potential (MEP) of **(a)** schiff base ligand (L) and its **(b)** Copper complexes (CuL), **(d)** Nickel complex (NiL) and **(c)** Cobalt complex (CoL).

MEP serves as a crucial tool for probing the electron density distribution within molecular systems, offering valuable insights into charge distribution and identifying potential reactive sites for electrophilic and nucleophilic attacks^63^. Furthermore, MEP is closely linked to key electronic properties of molecules, including polarizability and dipole moment, which can influence their reactivity and interaction profiles^64^. The MEP maps for the free ligand and its metal complexes are depicted in Fig. 10, revealing distinct electropositive and electronegative regions within the structures.

In the MEP map of the free ligand and its metal complexes, the electropositive areas (blue and green) and the electronegative areas (red and yellow) are distinctly marked. The negative regions, mainly located around the oxygen atoms of the –CONH groups and the nitrogen atoms of the C = NH groups, indicate possible sites for electrophilic attack. In contrast, the positive areas are primarily found around the hydrogen atoms of the C–H groups and the carbon atoms within the imine groups, pointing to likely nucleophilic centers. The MEP color scale of the free ligand ranges from − 0.110 a.u. (intense red) to + 0.110 a.u. (deep blue), highlighting a broad span of electrostatic potential. For the metal complexes CuL, NiL, and CoL, the MEP maps display potential ranges of − 0.253 a.u. to + 0.235 a.u. for CuL, − 0.222 a.u. to + 0.222 a.u. for NiL, and − 0.233 a.u. to + 0.233 a.u. for CoL. The color gradient progresses in the order red < orange < yellow < green < blue, representing increasing electrostatic potential. Although these ranges are slightly narrower than that of the free ligand, the pattern of electrostatic distribution remains consistent, with electronegative atoms showing negative regions and hydrogen and carbon atoms exhibiting positive zones. This indicates that the ligand’s electronegative atoms are key contributors in coordinating with metal centers, thereby affecting the complexes’ electronic structure and charge distribution. These electrostatic potential features directly influence molecular reactivity and biological interactions. Differences in MEP profiles between the ligand and its metal complexes highlight variations in electron-rich and electron-deficient regions, which govern how the molecules interact with nucleophilic or electrophilic sites in biological targets. This helps explain the observed differences in binding affinity and bioactivity among the complexes. As such, MEP analysis supports the potential of these compounds not only as therapeutic agents but also as molecular sensors and catalysts.

#### Frontier molecular orbitals (FMOs)

The Frontier Molecular Orbital (FMO) theory focuses on the Highest Occupied Molecular Orbital (HOMO) and the Lowest Unoccupied Molecular Orbital (LUMO), which are crucial for assessing a molecule’s chemical reactivity and kinetic stability. The HOMO reflects the molecule’s ability to donate electrons, while the LUMO represents its capacity to accept electrons. The energy gap, defined as the difference between the HOMO and LUMO energies (E_HOMO_ − E_LUMO_), provides valuable information about the molecule’s stability. A larger energy gap typically corresponds to greater stability and lower reactivity, whereas a smaller gap suggests higher reactivity, greater polarizability, and enhanced potential for biological interactions. A reduced HOMO–LUMO gap facilitates easier charge transfer between the molecule and a biological target, which may lead to stronger binding affinity and increased biological efficacy—particularly through mechanisms like hydrogen bonding, π–π stacking, and charge transfer^64,65^. These electronic characteristics are crucial for the biological efficacy of pharmacologically active compounds.

**Fig.11:**
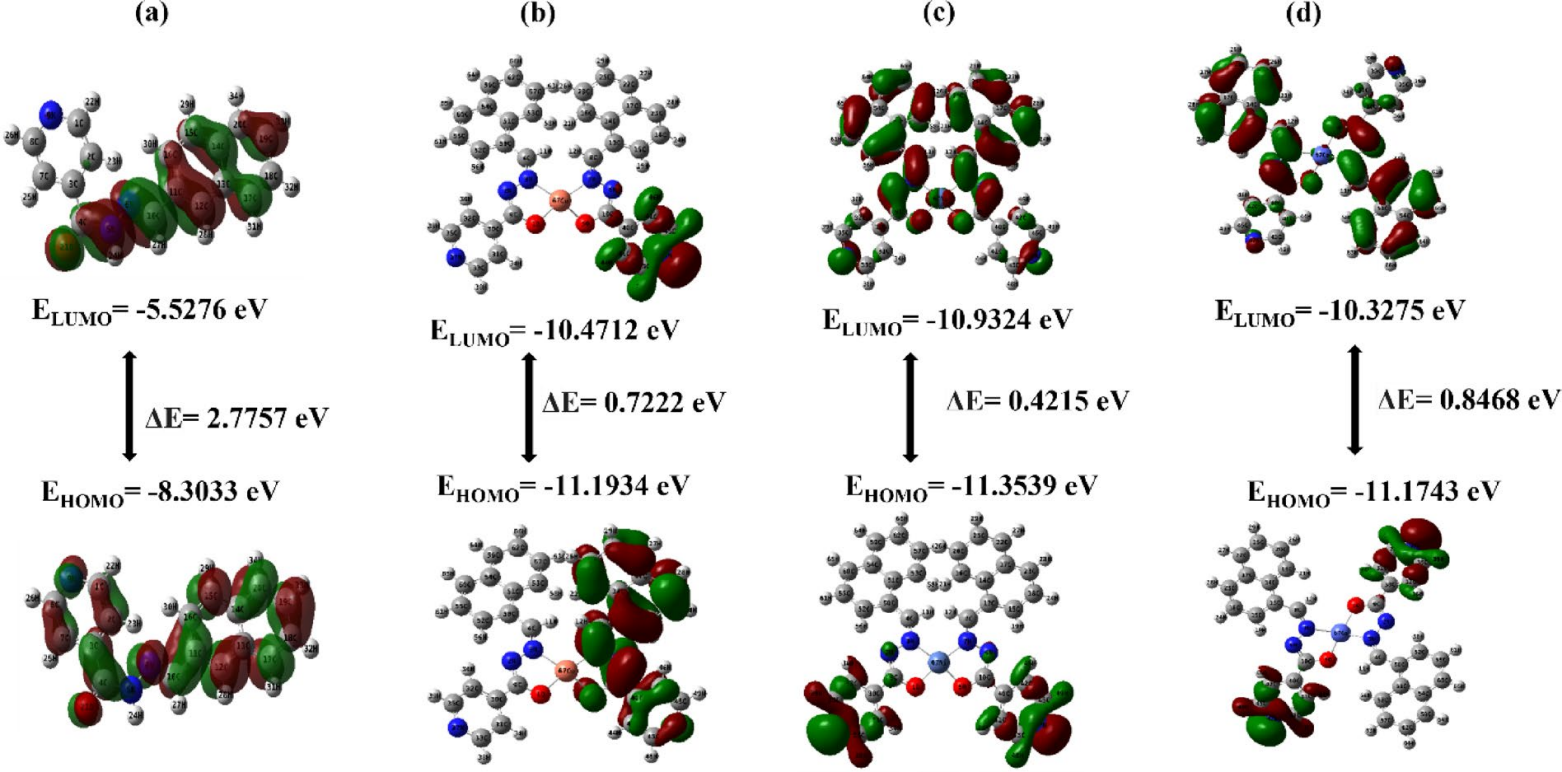
The difference between the E_HOMO_ and E_LUMO_ energy gap of the **(a)** schiff base ligand (L) and its **(b)** Copper complexes (CuL), **(d)** Nickel complex (NiL) and **(c)** Cobalt complex (CoL).

The key parameters obtained from this analysis are provided in Table 11, and the energy gap (ΔE) for the ligand and its metal complexes are illustrated in Fig. 11. These values offer a deeper understanding of the molecule’s behavior in various chemical reactions and its overall electronic characteristics.

**Table 11:** HOMO–LUMO gap and reactivity parameters of ligand and metal Complexes.

Name	E_HOMO_ (eV)	E_LUMO_ (eV)	Energy gap ΔE(eV)	Electron Affinity, A (eV)	Ionization energy, I_*p*_ (eV)	Hardness, η (eV)	Absolute softness, σ (eV)	Global Softness, S (eV)	Chemical potential, µ (eV)	Electronegativity, χ (eV)
L	−8.3033	−5.5276	2.7757	8.3033	5.5276	1.3879	0.7205	0.3603	−6.9155	6.9155
CuL	−11.1934	−10.4712	0.7222	11.1934	10.4712	0.3611	2.7693	1.3847	−10.8323	10.8323
NiL	−11.3539	−10.9324	0.4215	11.3539	10.9324	0.2108	4.7438	2.3719	−11.1431	11.1431
CoL	−11.1743	−10.3275	0.8468	11.1743	10.3275	0.4234	2.3618	1.1809	−10.7509	10.7509

The free schiff base ligand exhibited the highest energy gap (2.7757 eV), consistent with its poor antibacterial and antioxidant performance. Among the metal complexes, NiL showed the lowest energy gap (0.4215 eV), followed by CuL (0.7222 eV) and CoL (0.8468 eV). The ΔE of NiL suggests increased electronic reactivity and a greater ability to participate in charge transfer processes. This electronic feature facilitates stronger interactions with biomolecular targets, such as proteins or enzymes. The high reactivity associated with smaller energy gaps aligns with the superior biological performance of the NiL complex, as demonstrated by its highest docking score (–9.9 kcal/mol) and strongest antibacterial activity. Additionally, the d⁸ electronic configuration of Ni supports π-back bonding and enhancing stability^24^. These factors together explain the superior bioactivity of the NiL complex. In contrast, the relatively larger gaps in CuL and CoL may reduce its reactivity, contributing to its comparatively weaker biological effects. These results suggest that lower energy gaps may enhance the ability of the compounds to engage in non-covalent interactions within the active site of DNA gyrase, contributing to their overall therapeutic potential. This electronic structure-biological activity correlation has been similarly emphasized in recent computational studies involving schiff base systems^66^.

The electron affinity (A) values reveal that the metal centers enhance the ability of the complexes to accept electrons. The ligand has an electron affinity of 8.3033 eV, while the metal complexes show significantly higher values; reflecting enhanced electronic stabilization upon coordination. NiL, in particular, shows the highest electron affinity, highlighting its greater tendency to undergo reduction reactions^67^.

Ionization Potential (I_p_) reflects the energy required to remove an electron from a molecule, with higher values indicating greater stability and resistance to oxidation. The metal complexes have much higher ionization potentials compared to the ligand (5.5276 eV) suggesting that the metal centers help stabilize the complexes, making them less prone to oxidation. Regarding chemical hardness (η), which is half the energy gap, the ligand is harder and less reactive (η = 1.3879 eV) compared to the metal complexes, particularly NiL (η = 0.2108 eV), which is softer and more reactive. Other parameters, such as Absolute Softness (σ), Chemical Potential (µ), and Electronegativity (χ), further differentiate the reactivity of the compounds. NiL stands out with the highest Absolute Softness (4.7438 eV), Chemical Potential (−11.1431 eV), and Electronegativity (11.1431 eV), indicating it is the most reactive of the complexes. These findings demonstrate how the metal centers influence the electronic properties of the complexes, enhancing their reactivity while stabilizing them in certain aspects.

## Conclusion

In conclusion, this study successfully synthesized and characterized an isoniazid-derived schiff base ligand and its metal complexes with Cu(II), Ni(II), and Co(II) ions, aiming to develop compounds with potential antibacterial and antioxidant properties. Structural characterization confirmed successful complexation and stability of the compounds. The metal complexes, particularly those of nickel and copper, exhibited superior antibacterial and antioxidant activities compared to the free ligand. Molecular docking studies showed strong interactions between the complexes and a bacterial target protein, indicating their potential as therapeutic agents. Computational analyses further validated the stability of the complexes, revealing enhanced reactivity due to reduced energy gaps. Electron density mapping and partial charge analysis identified regions favorable for biological interactions. These findings suggest promising therapeutic potential. Future work will focus on in vivo evaluation of the most active complexes to validate their biological efficacy, along with structural modifications to enhance bioavailability and pharmacokinetic properties, thereby advancing their development as potential drug candidates in medicinal chemistry.

## Data Availability

Data are available to the corresponding author upon reasonable request.
